# High Intensity Interval Training Favourably Affects Angiotensinogen mRNA Expression and Markers of Cardiorenal Health in a Rat Model of Early-Stage Chronic Kidney Disease

**DOI:** 10.1155/2015/156584

**Published:** 2015-05-24

**Authors:** Patrick S. Tucker, Aaron T. Scanlan, Vincent J. Dalbo

**Affiliations:** ^1^Clinical Biochemistry Laboratory, Central Queensland University, Building 81, Bruce Highway, Rockhampton, QLD 4702, Australia; ^2^Human Exercise and Training Laboratory, Central Queensland University, Building 81, Bruce Highway, Rockhampton, QLD 4702, Australia

## Abstract

The majority of CKD-related complications stem from cardiovascular pathologies such as hypertension. To help reduce cardiovascular complications, aerobic exercise is often prescribed. Emerging evidence suggests high intensity interval training (HIIT) may be more beneficial than traditional aerobic exercise. However, appraisals of varying forms of aerobic exercise, along with descriptions of mechanisms responsible for health-related improvements, are lacking. This study examined the effects of 8 weeks of HIIT (85% VO_2max_), versus low intensity aerobic exercise (LIT; 45–50% VO_2max_) and sedentary behaviour (SED), in an animal model of early-stage CKD. Tissue-specific mRNA expression of RAAS-related genes and CKD-related clinical markers were examined. Compared to SED, HIIT resulted in increased plasma albumin (*p* = 0.001), reduced remnant kidney weight (*p* = 0.028), and reduced kidney weight-body weight ratios (*p* = 0.045). Compared to LIT, HIIT resulted in reduced* Agt* mRNA expression (*p* = 0.035), reduced plasma LDL (*p* = 0.001), triglycerides (*p* = 0.029), and total cholesterol (*p* = 0.002), increased plasma albumin (*p* = 0.047), reduced remnant kidney weight (*p* = 0.005), and reduced kidney weight-body weight ratios (*p* = 0.048). These results suggest HIIT is a more potent regulator of several markers that describe and influence health in CKD.

## 1. Introduction

The Australian Bureau of Statistics estimates that 14% of Australians (3.2 million) have at least one marker of kidney damage or dysfunction [1], putting them at an increased risk of developing chronic kidney disease (CKD). CKD is a progressive and irreversible condition, associated with an increased risk of developing cardiovascular disease (CVD) [2] and a decreased life expectancy [2]. It is possible to slow the progression of CKD, particularly while the disease is in its early stages, allowing for the delay of health-related complications and burdensome treatments associated with advanced CKD. However, as CKD progresses, CKD-related risk factors (e.g., hypertension) and comorbidities (e.g., CVD) become less manageable, resulting in a life expectancy that steadily decreases as a function of continual reductions in estimated glomerular filtration rate (eGFR) [2–4]. Considering the irreversible nature of CKD and the relationship between life expectancy and kidney function, it is apparent that slowing the progression of CKD is the ideal approach to CKD treatment.

As new and existing cases of CKD progress to the extent that they require renal replacement therapy (RRT), a term that refers to dialysis or renal transplantation, the existing burden on the healthcare system will increase considerably [5]. Direct financial impact will stem from increases in costly treatments such as dialysis, which is expensive compared to many medical treatments, while indirect financial impact will stem from an increased need for infrastructure including additional healthcare professionals, equipment, and treatment space [5]. While current rates of RRT are troubling (more than 20,000 Australians in 2012) [1], it is the pace at which RRT rates are increasing that is most concerning. Statistical analyses have indicated, by the year 2020, that RRT prevalence is expected to increase by 29% with an accompanying 37% increase in RRT-related expenditure [5]. The sharp increase in RRT is largely due to associated increases in risk factors including obesity [6], physical inactivity [6], and hypertension [7]. CKD-related hypertension is especially noteworthy as hypertension is closely associated with declining eGFR, independent of other risk factors [8, 9]. This hypertension-associated decline in eGFR is one reason, prior to commencing RRT; the most common CKD-related treatments focus on reducing hypertension. The rationale behind this approach is threefold: (1) CKD is an independent risk factor for cardiac events [10, 11]; (2) controlling hypertension is the best way to influence quality of life [12] and prognosis [13, 14] in CKD patients; (3) hypertension is the most influential modifiable-factor associated with CKD progression [15].

Central to the concept of hypertension is the renin angiotensin aldosterone system (RAAS), a general term applied to the network of enzymes and hormones that regulate blood pressure [16]. RAAS is characterised by renin, a protein encoded by the gene* Ren*, that cleaves a peptide bond on angiotensinogen, a protein encoded by the gene* Agt*, resulting in angiotensin I [17]. Although angiotensin I is biologically inactive on its own, it is converted to angiotensin II by angiotensin-converting enzyme [17], a protein encoded by the gene* Ace*. The resulting angiotensin II influences blood pressure by potentially binding to angiotensin II receptor, type 1 [17], a receptor encoded by the genes* Agtr1a *and* Agtr1b*, on intraglomerular mesangial cells, causing them to contract along with surrounding blood vessels. In addition, angiotensin II regulates the expression of* Cyp11b2*, a gene that encodes the enzyme aldosterone synthase, the enzyme that synthesises aldosterone [17]. Once synthesised, aldosterone promotes the reabsorption of sodium and water in the tubules of the kidney, leading to increased blood volume which results in increased blood pressure [18]. Considering this, it is apparent that intervention-based research, focused on modulating the activity/expression of the aforementioned RAAS-related compounds, is timely.

Chronic aerobic exercise training has emerged as a promising therapy in terms of reducing or regulating hypertension [19–21]. Considering that CKD progression is closely related to progressive hypertension [9, 22], chronic aerobic exercise training may be especially beneficial in patients with early-stage CKD who hope to avoid or delay the need for RRT. Recently, high intensity interval training (HIIT) (≈85% VO_2max_) has been the focus of many clinicians and researchers [23] who hypothesise that HIIT may elicit greater benefits, compared to traditional aerobic exercise (LIT) (≈50% VO_2max_) [24–26]. Reasons for this supposition include improved cardiovascular [24–26] and metabolic [23] outcomes. In addition, HIIT is also safe for many high-risk populations [26], including patients with CKD [27]. This, paired with the fact that HIIT encourages adherence to aerobic exercise training programs [28], makes HIIT an attractive therapeutic option for clinicians who aim to regulate blood pressure and delay the need for RRT in patients with CKD.

Although it has been established that aerobic exercise beneficially affects blood pressure, investigations that focus on the direct effects of HIIT on the molecular components of RAAS, especially in models of early-stage CKD, are unavailable. Moreover, the underlying molecular mechanisms that allow for increased benefit in HIIT, compared to traditional aerobic exercise, have yet to be described. This is largely due to the fact that studies conducting tissue-specific analyses of the molecular markers involved in RAAS are difficult to perform in humans, necessitating the need for animal models. As such, the purpose of the present study is to determine the effects of chronic HIIT (4 times per week for 8 weeks) on the mRNA expression of several key RAAS-related genes (*Ren*,* Agt*,* Ace*,* Agtr1a*,* Agtr1b*, and* Cyp11b2*), as well as traditional clinical markers of CKD (creatinine, albumin, total cholesterol, high-density lipoprotein, low-density lipoprotein, triglycerides, blood pressure, body weight, kidney weight, and kidney weight-body weight ratio), in an animal model of early-stage CKD. It was hypothesised that HIIT would exert a more beneficial effect on RAAS-related mRNA expression and traditional CKD-related clinical markers, compared to a sedentary (SED) condition and LIT.

## 2. Materials and Methods

### 2.1. Animals and General Overview

Male spontaneously hypertensive rats (SHR) (*n* = 39) (Animal Resource Centre, Canning Vale, WA, Australia) were housed in a temperature-controlled room (22–25°C) with a dark-light cycle of 12 : 12 hours and provided with* ad libitum* access to standard laboratory chow and water. At the time of arrival, rats were 4-5 weeks old. Following a 2-week habituation period, allowing for acclimatisation to their new environment, all rats underwent a unilateral nephrectomy (6-7 weeks old). Following surgery, rats were allowed to recover for 2 weeks (8-9 weeks old) prior to the initiation of an 8-week aerobic exercise intervention. Blood pressures, body weights, and body lengths were recorded at baseline (preexercise), 4 weeks (mid-intervention), and 8 weeks (postintervention). Following the 8-week intervention, rats were euthanised 24 hours following their last bout of exercise and tissues were collected for subsequent analysis (16-17 weeks old). All research procedures were granted prior approval by an institutional Animal Ethics Committee.

### 2.2. Anesthetisation and Euthanasia Procedures

Prior to nephrectomy, rats were anesthetised with Zoletil (25 mg/kg) and Xylazine (10 mg/kg). For pain relief, Meloxicam (2 mg/kg) was injected once daily for 2 days following nephrectomy. Prior to blood pressure readings, rats were anesthetised with Zoletil (25 mg/kg). Saline was injected subcutaneously following each anesthetisation to help avoid dehydration. Prior to being euthanised, rats were given a Lethabarb injection (1.5 mg/kg).

### 2.3. Disease Modelling

To mimic early-stage CKD, a reduction in renal function was induced by performing a unilateral nephrectomy. Briefly, a small lumbar incision was made and the left kidney was removed from each rat. In addition to nephrectomising the rats, SHRs were used to mimic the elevated blood pressure that is common in CKD. For more information regarding the disease model used in this study, as well as rationale, refer to Supplement 1 in Supplementary Material available online at http://dx.doi.org/10.1155/2015/156584.

### 2.4. Exercise Protocol

Rats were randomly assigned to one of three treatment groups: sedentary (SED, *n* = 12), light intensity aerobic exercise (LIT, *n* = 13), and high intensity interval aerobic exercise (HIIT, *n* = 14). The number of animals assigned to each group was dependent on the form of exercise, as the possibility existed that aerobic exercise may have proven too strenuous, resulting in the need to withdraw an animal from the experiment. However, there were no adverse events in any group, suggesting that aerobic exercise was well-tolerated in this disease model.

At 8-9 weeks of age, rats in the LIT and HIIT were introduced to treadmill running for 8 weeks. An identical 5-minute warm-up (15 m/min) was performed by both training groups before each exercise session. The LIT group exercised at a treadmill speed of 15 m/min with an incline of 1° (45–50% VO_2max_) [29], up to 33 minutes per day, 4 days per week, for 8 weeks. The HIIT group exercised at a treadmill speed of up to 50 m/min with an incline of up to 10° (≈85% VO_2max_) [29], 30 minutes per day (2-minute stationary period followed by 1-minute sprint × 10 repetitions), 4 days per week, for 8 weeks. Differing treadmill inclines were used to achieve the desired intensity. To permit adequate adaptation to treadmill running, thereby allowing each exercise session to be completed in its entirety, exercise training was progressive. By the beginning of the fourth week of training, all rats were performing exercise sessions at maximum intensity/duration; the exercise effort remained constant for the remaining 5 weeks of training. In an effort to standardise variables related to exercise training, while simultaneously allowing for the comparison of different forms, sessions between LIT and HIIT were matched based on distance travelled per bout. That is, despite the difference in exercise modality between groups, LIT and HIIT travelled the same distance (±1 meter) during each exercise session, with distances progressing in unison from ≈200 meters per session in week 1 to ≈500 meters per session in week 8 (Table 1).

### 2.5. Tissue Homogenisation and mRNA Isolation

Tissues used for analyses were chosen based on tissue-specific transcriptomic work performed in SHRs [30]. Renal tissue specifically consisted of renal cortex as mRNA is differentially expressed dependent on the type of renal tissue [31]. For the sake of consistency, hepatic tissue specifically consisted of the outer portion of the right lateral lobe.

Approximately 100 mg (±10 mg) of renal (for analysis of* Ren*,* Ace*,* Agtr1b*, and* Cyp11b2* mRNA) and hepatic (for analysis of* Agt* and* Agtr1a* mRNA) tissue was homogenised in 1 mL of TRI-reagent, using the TRI-reagent method (Sigma Aldrich, St. Louis, MO, USA). Samples were then centrifuged at 12,000 ×g for 10 minutes at 4°C. The resulting supernatant was transferred to a new microtube and incubated for 5 minutes at room temperature to allow for disassociation of the nucleoprotein complex. Following incubation, 0.2 mL of chloroform was added before this solution was mixed and then incubated for 3 minutes. Samples were then centrifuged at 12,000 ×g for 15 minutes at 4°C. The resulting supernatant was transferred to a new microtube. Following this separation, 500 *μ*L of 2-propanol was added to each microtube. Microtubes were incubated at room temperature for 10 minutes and then centrifuged at 12,000 ×g for 10 minutes at 4°C. The resulting supernatant was removed, leaving behind a mRNA pellet. The mRNA pellet was exposed to 2 ethanol washes (1 mL, 75%). Following each wash, microtubes were vortexed and centrifuged at 7,500 ×g for 5 minutes at 4°C. The mRNA pellets were then allowed to air dry under a fume hood before being dissolved in 30 *μ*L of RNase-free water. A 260/280 ratio was calculated to assess mRNA purity, with all ratios falling within commonly accepted parameters (260/280 ratio of 1.9–2.1) (NanoDrop 2000c, Thermo Scientific, Wilmington, DE, USA). The diluted mRNA samples were stored at −80°C until later analyses.

### 2.6. Total mRNA Determination and Reverse Transcription

Total mRNA concentrations from each sample were determined using a spectrophotometer (NanoDrop 2000c, Thermo Scientific, Wilmington, DE, USA). Next, mRNA concentrations were adjusted to 400 ng/*μ*L by diluting the crude total mRNA extracts with RNase-free water. The standardised solutions of total cellular mRNA were reverse transcribed to synthesise complimentary DNA (cDNA) using a High Capacity cDNA Reverse Transcription Kit (Applied Biosystems, Foster City, CA, USA). Briefly, 2x RT master mix was prepared per kit instructions. Next, 10 *μ*L of RT mix was added (and mixed via pipette trituration) to 10 *μ*L of the standardised mRNA solution. These samples were placed on a thermal cycler (T100 Thermal Cycler, Bio-Rad, Gladesville, NSW, Australia) set to run at 25°C for 10 minutes, 37°C for 120 minutes, and 85°C for 5 minutes. Following reverse transcription, the resulting cDNA template concentrations were diluted to 5 ng/*μ*L by adding RNase-free water. The standardised cDNA solutions were frozen at −80°C until quantitative reverse transcription polymerase chain reaction (qRT-PCR) was performed.

### 2.7. Quantitative Reverse Transcription Polymerase Chain Reaction Analysis

Forward and reverse oligonucleotide primer pairs were developed using National Centre for Biotechnology Information's (NCBI, Bethesda, MD, USA) Primer Designer Tool before being commercially synthesised (GeneWorks, Hindmarsh, SA, Australia) (Table 2).* Gapdh* was used as an internal reference gene for detecting relative change in the quantity of target mRNA due to its consideration as a constitutively expressed housekeeping gene following aerobic exercise training. Following the examination of primer efficiencies, qRT-PCR reactions were mixed. Reactions contained the following mixture: 2.5 *μ*L of prepared cDNA template, 5 *μ*L of SYBR Select Master Mix (Life Technologies, Mulgrave, Victoria, Australia), 0.5 *μ*L of sense and antisense primers, and 1.5 *μ*L RNase-free water. Following the mixture of each reaction, qRT-PCR was performed with a thermal cycler (Rotor-Gene Q, Qiagen, Venlo, Netherlands). The amplification sequence involved an initial 10-minute cycle at 95°C, followed by 40 cycles, each composed of a denaturation step (95°C for 15 seconds) and a primer annealing/extension step (60°C for 45 seconds). Melting curves were performed to ensure that sufficient PCR product was being generated by each primer, in the absence of primer dimers. All assays were performed in duplicate (coefficient of variation = 0.01). mRNA expression data were calculated using the Livak method [32] and data are presented as the fold change of the gene of interest, relative to that of control animals (SED).

### 2.8. Clinical Markers in Plasma

All blood/plasma markers were assessed using blood taken from the descending aorta of each rat. Blood was collected into EDTA tubes, following lethal injection, directly after the thoracic cavity of the animal had been opened, before tissues were collected. This blood was centrifuged for 10 minutes at 1500 ×g and the resulting plasma was aliquoted to 2.0 mL microtubes and frozen at −80°C until it was analysed.

Plasma triglycerides were measured using a commercially available assay kit (Abcam, Cambridge, England, United Kingdom). Briefly, standards were prepared per the kit instructions. Next, 25 *μ*L of plasma was combined with assay buffer in the well of a 96-well plate. Lipase (2 *μ*L) was added to each well and solutions were mixed before incubating for 20 minutes at room temperature. Triglyceride reaction mix was added to each well and solutions were mixed before incubating for 60 minutes at room temperature. Triglyceride concentrations were measured colorimetrically.

Plasma high-density lipoprotein (HDL) and low-density lipoprotein (LDL) were measured using a commercially available assay kit (Abcam, Cambridge, England, United Kingdom). HDL and LDL fractions of plasma samples were separated per the kit instructions and standards were prepared. Next, 15 *μ*L of HDL and LDL fractions was first combined with cholesterol assay buffer, followed by reaction mix. Solutions were mixed and then allowed to incubate for 60 minutes at 37°C. HDL and LDL concentrations were measured colorimetrically. Total cholesterol was calculated using a variation of the Friedlander formula [33].

Plasma creatinine was measured using a commercially available assay kit (Abcam, Cambridge, England, United Kingdom). Standards were prepared per the kit instructions. Next, 25 *μ*L of plasma was combined with assay buffer in the well of a 96-well plate. Reaction mix was added to this solution and samples were allowed to incubate at 37°C for 60 minutes. Creatinine concentrations were measured colorimetrically.

Plasma albumin was measured using a commercially available enzyme-linked immunosorbent assay (ELISA) kit (Abcam, Cambridge, England, United Kingdom). Standards were prepared per the kit instructions. Plasma samples were prepared and added to sample wells. Biotinylated albumin was then added to each well and this solution was mixed gently before a 60-minute incubation period. Following several washes, 1x Streptavidin-Peroxidase (SP) conjugate was added to each well prior to a 30-minute incubation period. Once again, plates were washed and 50 *μ*L of chromogen substrate was added, followed by a 12-minute incubation period. Stop solution was then added to each well and albumin concentrations were measured colorimetrically.

### 2.9. Clinical and Anthropometric Measurements

Blood pressures, body weights, and body lengths were recorded before surgery, baseline (preexercise), 4 weeks (mid-intervention), and 8 weeks (postintervention). Kidney weights were recorded after intervention. Systolic blood pressure (SBP) was measured using data acquisition hardware (PowerLab, ADInstuments, Bella Vista, NSW, Australia) coupled to a noninvasive blood pressure (NIBP) system (NIBP System, ADInstuments, Bella Vista, NSW, Australia) with a pulse transducer/cuff (Pulse Transducer/Pressure Cuff for NIBP, ADInstuments, Bella Vista, NSW, Australia). Measurements were then analysed using LabChart 8 (ADInstuments, Bella Vista, NSW, Australia). Body length was measured from tip-of-nose to tip-of-tail. Body weight and remnant kidney weight were measured to the nearest thousandth of a gram. Body length and body weight measurements were taken while each rat was sedated, directly prior to SBP measurement.

### 2.10. Statistics

mRNA data are presented as mean ± standard error of the mean (SEM). All other data are presented as mean ± standard deviation (SD). Body length, body weight, body length-body weight ratio, and SBP data were analysed using 3 × 3 (group × time) repeated measures analysis of variance (RMANOVA). When applicable, group effects and/or time effects were examined using separate one-way ANOVAs with Tukey post hoc comparisons. Data for all other measures (mRNA expression, plasma markers, kidney weight, and kidney weight-body weight ratio) were analysed using one-way ANOVAs with Tukey post hoc comparisons. Analyses were performed using IBM SPSS Statistics (v20.0, IBM Corporation; Armonk, NY, USA). Statistical significance was set at *p* < 0.05.

## 3. Results

### 3.1. Quantitative Reverse Transcription Polymerase Chain Reaction Analysis

**Figure 1:**
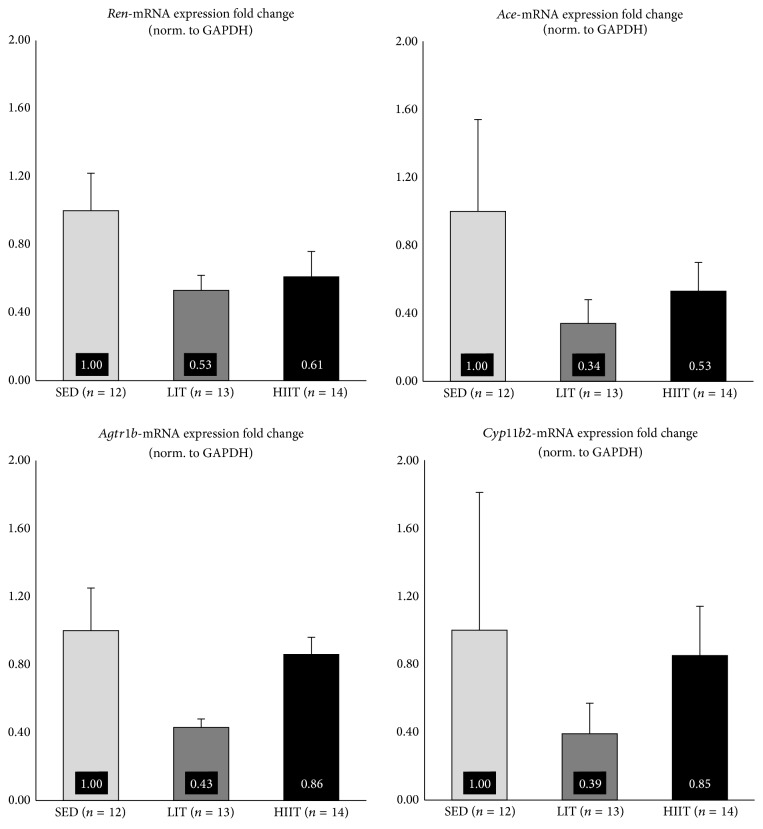
Renal mRNA expression fold change.

**Figure 2:**
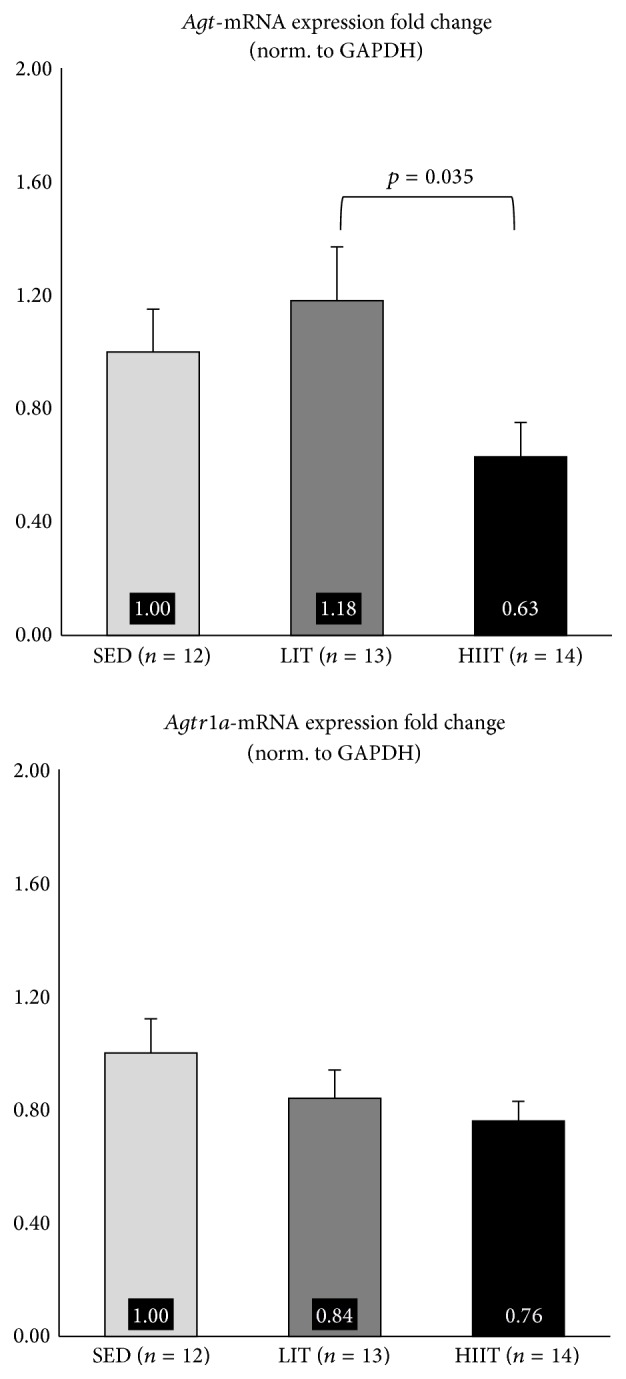
Hepatic mRNA expression fold change.

Results pertaining to renal expression of RAAS-related mRNA can be seen in Figure 1. Results pertaining to hepatic expression of RAAS-related mRNA can be seen in Figure 2. Expression of renal* Ren, Ace, Agtr1b, and Cyp11b2* mRNA and hepatic* Agtr1a* mRNA was not significantly different between groups (*p* > 0.05). Expression of hepatic* Agt* mRNA was significantly decreased in HIIT, compared to LIT (*p* = 0.035).

### 3.2. Clinical Markers in Plasma

Results pertaining to clinical markers in plasma can be seen in Table 3. Plasma measures LDL (*p* = 0.001), triglycerides (*p* = 0.029), and total cholesterol (*p* = 0.002) were significantly lower in HIIT, compared to LIT. Plasma albumin was significantly higher in HIIT, compared to SED (*p* = 0.001) and LIT (*p* = 0.047).

### 3.3. Anthropometric Measurements and Kidney Weight

Results pertaining to anthropological measurements and kidney weight can be seen in Tables 3 and 4. Body Length, body weight, body length-body weight ratio, and SBP increased significantly from baseline to 4 Weeks and from 4 Weeks to 8 Weeks (*p* < 0.05) in all groups (Table 4), with no significant group interactions. Kidney weight was significantly lower in HIIT, compared to the SED (*p* = 0.028) and LIT (*p* = 0.005). Kidney weight-body weight ratio was significantly lower in HIIT, compared to the SED (*p* = 0.045) and LIT (*p* = 0.048) (Table 3).

## 4. Discussion

The current study examined the ability of varying forms of aerobic exercise to influence mRNA expression of several RAAS-related genes, as well as common CKD-related clinical and anthropometric markers, in an animal model of early-stage CKD. This is the first investigation to compare the efficacy of varying forms of aerobic exercise as it pertains to the modulation of tissue-specific mRNA expression of RAAS-related genes. HIIT produced more favourable outcomes, compared to SED and LIT, in that HIIT resulted in significantly reduced mRNA expression of* Agt* (compared to LIT), significantly improved plasma levels of LDL, triglycerides, and total cholesterol (compared to LIT), significantly improved plasma levels of albumin (compared to SED and LIT), and significantly reduced remnant kidney weight and kidney weight-body weight ratios (compared to SED and LIT).

Results regarding the mRNA expression of* Agt* are interesting. The mRNA expression of all other genes (*Ace, Ren, Agtr1a, Agtr1b*, and* Cyp11b2*) was nonsignificantly downregulated in both exercise groups, compared to SED. However,* Agt* mRNA expression was upregulated in LIT, compared to SED (not significant) and HIIT (*p* = 0.035). It is worth mentioning that our results are not the first data to suggest that lower intensity aerobic exercise training may result in unfortunate adaptations in animal models of CKD; aerobic exercise training performed at 50–70% of VO_2max_ has been reported to result in increased resting SBP and decreased myocardial capillary density, in an animal model of CKD [34].

Differential mRNA expression of* Agt* may partially explain the nonsignificant (*p* = 0.074) difference in SBP in HIIT during Week 8, compared to SED (*p* = 0.14, +5.28%) and LIT (*p* = 0.74, +6.01%). The downregulation in the mRNA expression of* Agt* in HIIT may also explain the lack of significant differences in other RAAS genes in this study. That is, a decrease in the mRNA expression of* Agt* may have quenched the need for any additional significant decreases in the expression of other RAAS genes. This may explain why, even though there was a near-significant reduction in SBP in HIIT compared to LIT (*p* = 0.074), mRNA expression of several RAAS-related of genes (*Ace, Ren, Agtr1b*, and* Cyp11b2*) was slightly, but not significantly, increased in HIIT compared to LIT. A review on hepatic angiotensinogen [35] discusses the noteworthy influence that angiotensinogen has on blood pressure. Decreased levels of angiotensinogen using angiotensinogen-antibodies in rats [36] and* Agt*-knockout mice [37] exhibit lower blood pressure, relative to controls. Conversely, angiotensinogen infusion in mice [38] and* Agt* overexpression in rats [39] result in increased blood pressure. Evidence suggests that angiotensinogen is a more potent influencer of blood pressure than previously suspected [35, 39], often more influential than other components of RAAS [40], and deserves more attention as a potential target in hypertension-related therapies [35], proposals that are supported by our findings.

Large amounts of between-animal variation in the mRNA expression of RAAS-related genes in SED may explain the lack of significant results in the current study. Duplicate-duplicate variation was low (CV = 0.01), suggesting large amounts of between-animal variation in SED. The issue of between-animal mRNA variation has been empirically addressed in the past [41–43], with the most commonly suggested remedy being an increase in sample size. Interestingly, variation in between-animal mRNA expression in SED is higher across all genes (except for* Agt*), compared to LIT and HIIT, similar to previous work that has examined RAAS-related mRNA expression in sedentary versus exercise-trained SHRs [20, 44]. This, combined with the fact that aerobic exercise resulted in a downregulation across all genes, compared to SED (expect for* Agt* in LIT), suggests that RAAS-related mRNA expression may be poorly and indiscriminately regulated, in the absence of aerobic exercise, in an animal model of early-stage CKD. That is, aerobic exercise may help to control otherwise haphazard RAAS-related mRNA expression that occurs during the early stages of CKD. Several informative reviews have outlined the issue of unregulated blood pressure control [45] and, more specifically, unregulated RAAS activity [46] in CKD, but this is the first study to suggest that one of the mechanisms by which aerobic exercise may prove beneficial is via tightened control of RAAS-related mRNA expression, independent of statistically significant improvements to RAAS-related mRNA expression.

In the current study, the significant decreases in plasma LDL, triglycerides, and total cholesterol in HIIT, compared to LIT but not SED, are encouraging and somewhat surprising. A recent study performed in healthy, well-trained humans reported nonsignificant improvements in LDL, triglycerides, and total cholesterol after 12 weeks of HIIT [47]. In special and/or clinical populations, HIIT seems to exert more influence over blood lipids. Cardiac rehabilitation patients who engaged in HIIT experienced significant improvements in HDL, relative to traditional aerobic exercise training [26]. However, triglyceride measurements were not different between groups and LDL measurements were not reported [26]. HIIT has also proven to be beneficial in obese men [48] and women [49], resulting in improved HDL [48], LDL [48], triglycerides [48, 49] and total cholesterol [48, 49]. Despite the slowly-accumulating data, a recent review [50] that notes the ability of HIIT to influence blood lipids has yet to be established, especially in special populations, suggesting that this specific line of research, the effects of HIIT on blood lipids, should be further examined. This is especially true in CKD as lipid-related disorders are the leading cause of complications in CVD [51, 52] and CVD-related complications are the leading cause of death in CKD patients [10, 11]. As such, dyslipidemia should be ranked among the primary concerns of CKD-related research [53].

Similar to blood lipids, a recent review notes that the effects of exercise training on plasma albumin in patients with CKD are unclear [54]. Moreover, data describing the effects of HIIT on plasma albumin in patients with CKD are unavailable. Hypoalbuminemia is an independent predictor of renal dysfunction [55] and an independent predictor of CVD in early-stage CKD [56] and can be used to identify CKD patients at increased risk of developing CVD [56]. In obese individuals with metabolic syndrome, two common characteristics of CKD, 12 weeks of aerobic exercise training significantly reduced albuminuria (urinary albumin), compared to a no-exercise control group [57]. Aerobic exercise training (16 weeks at 60% of maximal aerobic velocity) has also proven useful at reducing albuminuria in SHRs [44]. Because hypoalbuminemia is related to declining kidney function [55], blood albumin and/or urinary albumin are useful markers for assessing the preservation of renal tissue. In the current study, plasma albumin was significantly higher in HIIT, compared to SED and LIT, suggesting that renal structure and function are better preserved by HIIT. The preservation of renal structure and function, and subsequent increase in plasma albumin, may have been due to the downregulation of* Agt* mRNA expression and the accompanying decrease in SBP in HIIT, as hypertension is directly related to renal damage [44, 58].

Also considered an indicator of renal damage, kidney weight and kidney weight-body weight ratio were significantly lower in HIIT, compared to SED and LIT. Hypertension-related declines in renal function are associated with compensatory alterations to renal tissue such as tubulointerstitial fibrosis [59, 60]. Often pathological, these alterations result in glomerular hypertrophy [59, 60]. Using histological and molecular applications, 16 weeks of aerobic exercise training (performed at 60% of maximal aerobic velocity) has been shown to reduce renal fibrosis in SHRs [44]. Although renal fibrosis and renal hypertrophy are directly related, data that specifically describes the effects of HIIT on renal hypertrophy are unavailable. Similar to the mechanisms that account for increased levels of plasma albumin in HIIT, there is evidence to suggest that the significantly lower kidney weight and kidney weight-body weight ratios in HIIT, compared to SED and LIT, may be due to decreased fibrosis. Decreases in renal fibrosis may be due to decreases in hypertension-mediated renal damage following a downregulation of the mRNA expression of* Agt* and a decrease in SBP (in HIIT) [44, 58].

## 5. Conclusions

In summary, in an animal model of early-stage CKD, HIIT appeared to be more beneficial, compared to SED and LIT, as HIIT was responsible for a significant downregulation in the mRNA expression of* Agt*. The downregulated mRNA expression of* Agt*, along with significant improvements to blood lipids (LDL, triglycerides, and total cholesterol), may partially explain the nonsignificant decrease in SBP and significant improvements to plasma albumin, kidney weight, and kidney weight-body weight ratios in HIIT, relative to SED and LIT. Future work would benefit from examining RAAS-related protein expression as well as specific measures of renal histology and morphology. Future work would also benefit from examining whether the positive HIIT-related effects, seen in the present study, are apparent in different related disease models (e.g., nonnephrectomised SHRs or 5/6 nephrectomy SHRs).

## Supplementary Material

Supplement 1 provides detailed information regarding the disease characteristics apparent in the early-stage CKD model used in this study. In addition, Supplement 1 provides rationale for the use of this disease model. It is the hope of the authors that the consolidation of this information will be useful to other researchers in this area.

## Figures and Tables

**Table 1:** Detailed exercise protocol.

Group	Variable	Measure	Wk 1	Wk 2	Wk 3	Wk 4	Wk 5	Wk 6	Wk 7	Wk 8
SED	NA	NA	NA	NA	NA	NA	NA	NA	NA	NA

LIT 45–50% VO_2max_	Max. speed	Meters/Min	15	15	15	15	15	15	15	15
	Incline	Degrees	1	1	1	1	1	1	1	1
	Duration	Minutes	13.3	20	26.6	33.3	33.3	33.3	33.3	33.3
	Intensity	Constant treadmill speed and constant treadmill incline								
	Frequency	Bouts/Wk	4	4	4	4	4	4	4	4
	Distance per bout	Meters	199.5	300	399	499.5	499.5	499.5	499.5	499.5
	Distance per week	Meters	798	1200	1596	1998	1998	1998	1998	1998

HIIT ≈85% VO_2max_	Max. speed	Meters/Min	20	30	40	50	50	50	50	50
	Incline	Degrees	5	10	10	10	10	10	10	10
	Duration	Minutes	30	30	30	30	30	30	30	30
	Intensity	2 min stationary period followed by 1 min sprint period × 10 reps								
	Frequency	Bouts/Wk	4	4	4	4	4	4	4	4
	Distance per bout	Meters	200	300	400	500	500	500	500	500
	Distance per week	Meters	800	1200	1600	2000	2000	2000	2000	2000

Identical 5-min warm-up (15 m/min) was performed by both training groups prior to each exercise bout.

**Table 2:** Primer sequences.

Target	Primer sequence (5′-3′)
Angiotensin I converting enzyme (Ace) forward	CTTGACCCTGGATTGCAGCC
Angiotensin I converting enzyme (Ace) reverse	GTTTCGTGAGGAAGCCAGGA
Angiotensin II receptor, type 1a (Agtr1a) forward	AGTCCTGTTCCACCCGATCA
Angiotensin II receptor, type 1a (Agtr1a) reverse	TCCAGACAAAATGCCAGCCA
Angiotensin II receptor, type 1b (Agtr1b) forward	ACTGCACACGGTGCATTTTA
Angiotensin II receptor, type 1b (Agtr1b) reverse	TAATTGTGCCTGCCAGCCTT
Angiotensinogen (serpin peptidase inhibitor, clade A, member 8) (Agt) forward	CTGGAGCTAAAGGACACACAGA
Angiotensinogen (serpin peptidase inhibitor, clade A, member 8) (Agt) reverse	GTGAAGGGACCCAAGCTCTC
Cytochrome P450, family 11, subfamily b, polypeptide 2 (Cyp11b2) forward	TAACTCAGGGAGCTTTACCTCT
Cytochrome P450, family 11, subfamily b, polypeptide 2 (Cyp11b2) reverse	CTGAGACCCTTTGAAGGCCG
Renin (Ren) forward	CCGTGGTCCTCACCAACTAC
Renin (Ren) reverse	CTTGGCCAGCATGAAGGGTA
Glyceraldehyde-3-phosphate dehydrogenase (Gapdh) forward	GTTACCAGGGCTGCCTTCTC
Glyceraldehyde-3-phosphate dehydrogenase (Gapdh) reverse	GATGGTGATGGGTTTCCCGT

**Table 3:** Nonrepeated clinical and anthropometric measures.

Clinical measure	Group	Mean	Standard deviation	Mean duplicate CV
HDL (mg/mL)	SED	0.82	0.13	0.06
	LIT	0.88	0.15	
	HIIT	0.93	0.12	

LDL (mg/mL)	SED	1.27	0.27	0.03
	LIT	1.55	0.34	
	HIIT	1.08^∗^	0.32	

TAG (mg/mL)	SED	5.65	1.66	0.04
	LIT	6.35	2.65	
	HIIT	4.11^∗^	2.04	

CHOL (mg/mL)	SED	3.22	0.47	NA
	LIT	3.71	0.61	
	HIIT	2.83^∗^	0.73	

CRE (nmol/*μ*L)	SED	1.07	0.51	0.04
	LIT	2.06	1.79	
	HIIT	2.11	1.26	

ALB (mg/mL)	SED	26.23	3.84	0.04
	LIT	29.88	6.42	
	HIIT	35.24^†^	6.08	

Kidney weight (g)	SED	1.57	0.09	NA
	LIT	1.59	0.12	
	HIIT	1.46^†^	0.08	

KW-BW ratio	SED	0.0050	0.0003	NA
	LIT	0.0050	0.0003	
	HIIT	0.0047^†^	0.0002	

Significance set at *p* ≤ 0.05.

^∗^Significantly different from LIT.

^†^Significantly different from LIT and SED.

HDL: high density lipoprotein; LDL: low density lipoprotein; TAG: triglycerides; CHOL: total cholesterol; CRE: creatinine; ALB: albumin; KW-BW: kidney weight-body weight; CV: coefficient of variation.

**Table 4:** Repeated clinical and anthropometric measures.

Clinical measure	Group	Baseline		4 Weeks^∗^		8 Weeks^†^	
		(Preintervention)		(Mid-Intervention)		(Postintervention)	
Body length (cm)	SED	28.48	(1.86)	35.03	(0.85)	37.41	(1.06)
	LIT	29.32	(1.65)	35.15	(0.79)	37.89	(0.88)
	HIIT	29.61	(2.25)	35.46	(0.65)	37.87	(0.62)

Body weight (g)	SED	224.42	(17.82)	276.05	(18.31)	313.53	(16.62)
	LIT	231.59	(21.01)	277.42	(20.77)	318.25	(21.03)
	HIIT	230.90	(19.98)	274.87	(16.46)	308.16	(17.58)

BL-BW ratio	SED	0.126	(0.007)	0.127	(0.007)	0.120	(0.004)
	LIT	0.127	(0.009)	0.128	(0.007)	0.119	(0.009)
	HIIT	0.129	(0.007)	0.130	(0.008)	0.124	(0.006)

Systolic blood pressure (mmHg)	SED	175.57	(9.27)	207.77	(17.39)	239.77	(12.85)
	LIT	173.09	(9.45)	209.25	(23.1)	241.63	(21.42)
	HIIT	174.65	(14.82)	216.25	(16.24)	227.12	(14.23)

Values presented as mean (standard deviation).

Significance set at *p* ≤ 0.05.

^∗^Significantly different compared to baseline, for all groups.

^†^Significantly different compared to baseline and 4 weeks, for all groups.

BL-BW: body length-body weight.
