# Supplementary material for: High Intensity Interval Training Favourably Affects Angiotensinogen mRNA Expression and Markers of Cardiorenal Health in a Rat Model of Early-Stage Chronic Kidney Disease
Source: Biomed Res Int. 2015 May 24;2015:156584. doi: 10.1155/2015/156584 (PMC4458272; doi:10.1155/2015/156584)
Supplement: Supplementary file 1 — Supplement 1 provides detailed information regarding the disease characteristics apparent in the early-stage CKD model used in this study. In addition, Supplement 1 provides rationale for the use of this disease model. It is the hope of the authors that the consolidation of this information will be useful to other researchers in this area. [file 156584.f1.pdf]

Supplement 1. Disease Modelling – Background and Rationale

To mimic early-stage CKD, renal function was artificially reduced by performing a unilateral nephrectomy. Briefly, a small lumbar incision was made and the left kidney was removed from each rat. Unilateral nephrectomies are an appropriate model of early-stage CKD in rats, as opposed to the commonly used 5/6 nephrectomy which more closely models end-stage renal disease. Unilateral nephrectomies result in compensatory enlargement of the remnant kidney [61-65], increased serum creatinine concentrations [63, 66, 67], increased focal glomerulosclerosis [68, 69], increased mesangial sclerosis [68], increased interstitial fibrosis [68], increased proteinuria [66, 68, 70], and increased urinary albumin excretion [69]. In addition to nephrectomising the rats, SHRs were used to mimic the elevated blood pressure that is common in CKD. In male SHRs, that have not undergone a nephrectomy, systolic blood pressure consistently increases at a rate of approximately 3.5 mmHg per week beginning at approximately 8 weeks of age, the initiation of intervention in the present study, plateauing at roughly 16 weeks of age, the termination of intervention in the present study [71, 72]. In addition, SHRs have fewer glomeruli than other strains [73], resulting in a reduced glomerular volume which is thought to influence blood pressure, making SHRs an appropriate model for hypertension- and CKD-related research.

References

61. Benipal, B. and L.H. Lash, *Modulation of mitochondrial glutathione status and cellular energetics in primary cultures of proximal tubular cells from remnant kidney of uninephrectomized rats.* Biochem Pharmacol, 2013. **85**(9): p. 1379-88.
62. Lopez-Novoa, J.M., et al., *Functional compensatory changes after unilateral nephrectomy in rats. General and intrarenal hemodynamic alterations.* Ren Physiol, 1982. **5**(2): p. 76-84.
63. Orsic, V., et al., *Impaired kidney function in rats six months after unilateral nephrectomy - an old story, a new perspective.* Med Glas (Zenica), 2011. **8**(2): p. 185-91.
64. Provoost, A.P. and J.C. Molenaar, *Changes in the glomerular filtration rate after unilateral nephrectomy in rats.* Pflugers Arch, 1980. **385**(2): p. 161-5.
65. Shirley, D.G. and S.J. Walter, *Acute and chronic changes in renal function following unilateral nephrectomy.* Kidney Int, 1991. **40**(1): p. 62-8.
66. Rodriguez-Gomez, I., et al., *Long-term consequences of uninephrectomy in male and female rats.* Hypertension, 2012. **60**(6): p. 1458-63.
67. Steffes, M.W., D.M. Brown, and S.M. Mauer, *Diabetic glomerulopathy following unilateral nephrectomy in the rat.* Diabetes, 1978. **27**(1): p. 35-41.
68. Adler, S., et al., *Studies of progressive glomerular sclerosis in the rat.* Am J Pathol, 1986. **123**(3): p. 553-62.
69. Celsi, G., S.O. Bohman, and A. Aperia, *Development of focal glomerulosclerosis after unilateral nephrectomy in infant rats.* Pediatr Nephrol, 1987. **1**(3): p. 290-6.
70. Simons, J.L., et al., *Pathogenesis of glomerular injury in the fawn-hooded rat: effect of unilateral nephrectomy.* J Am Soc Nephrol, 1993. **4**(6): p. 1362-70.
71. Cejka, J., et al., *LVV-hemorphin-7 lowers blood pressure in spontaneously hypertensive rats: radiotelemetry study.* Physiol Res, 2004. **53**(6): p. 603-7.
72. Firbas, U., W. Michlits, and S. Aharinejad, *Venous sphincters in the choroidea of spontaneously hypertensive rats.* Anat Rec A Discov Mol Cell Evol Biol, 2005. **284**(2): p. 537-43.
73. Black, M.J., et al., *Is there an association between level of adult blood pressure and nephron number or renal filtration surface area?* Kidney Int, 2004. **65**(2): p. 582-8.
